# Development and application of an ANN-perception-based autonomous control system for *Escherichia coli* cultivation process

**DOI:** 10.3389/fmicb.2026.1791815

**Published:** 2026-03-09

**Authors:** Mengxuan Zhou, Beichen Zhao, Zhiren Gan, Jingyan Jiang, Renquan Guo, Nikolai Mushnikov, Xueliang Li, Jian Ding, Zhenggang Xie

**Affiliations:** 1 Key Laboratory of Industrial Biotechnology, Ministry of Education, School of Biotechnology, Jiangnan University, Wuxi, China; 2 Department of Chemical and Materials Engineering, The University of Auckland, Auckland, New Zealand; 3 T&J Bio-Engineering (Shanghai) Co., Ltd., Shanghai, China; 4 Department of Biosciences, Faculty of Sciences, University Technology Malaysia, Johor, Malaysia

**Keywords:** ANN model, autonomous control, DO-stat, *Escherichia coli*, sfGFP

## Abstract

To address the challenges of overflow metabolism and the heavy reliance on manual intervention in high-density *Escherichia coli* fermentation, this study introduces an AI-driven, autonomous intelligent control system. Using superfolder green fluorescent protein (sfGFP) as a reporter, the research first optimized DO-stat feeding parameters and the induction process, achieving a 52.85% increase in cellular specific fluorescence intensity and significantly enhancing protein expression levels. Subsequently, an artificial neural network (ANN) model was developed and trained to achieve real-time recognition of dissolved oxygen (DO) baselines (*R*^2^ = 0.998). This model was integrated with feeding control logic to form the NeuroStat-Ctrl system, enabling fully autonomous control across the entire fermentation lifecycle. Utilizing this system, unattended *E. coli* fermentation was successfully achieved, with fluorescent protein production further increasing by 5.87% compared to the optimized manual control. Experimental validation demonstrated that the system effectively mitigates feeding deviations inherent in traditional fixed-threshold strategies, prevents metabolic overflow, and enhances process stability and reproducibility. Furthermore, this system provides an efficient, standardized, and intelligent solution for high-throughput strain screening and process validation in parallel bioreactors.

## 1 Introduction

*Escherichia coli (E. coli)* remains the predominant host for recombinant protein production, favored for its rapid growth kinetics, well-characterized genetic background, and high productivity (Gopal and Kumar, 2013; Rosano and Ceccarelli, 2014). To maximize high cell density and protein yield, numerous studies have employed methods such as medium optimization, precise dissolved oxygen control, and fed-batch cultivation (Riesenberg and Guthke, 1999; Zhou et al., 2018; Datta et al., 2021). However, high-density cultivation is often limited by the phenomenon of “overflow metabolism.” When the glucose supply exceeds the consumption capacity of the bacteria, carbon metabolic flux shifts towards the production of inhibitory by-products (such as acetate), even under conditions of sufficient oxygen supply. Acetate accumulation not only inhibits cell growth but also affects the synthesis and correct folding of recombinant proteins (Waegeman and Soetaert, 2011). Therefore, employing a restricted feeding strategy to maintain substrate concentration below a critical threshold is crucial for high yields of recombinant proteins.

DO-stat is a common feeding strategy that maintains substrate concentration at a critical value and is widely applied due to its operational simplicity and sensitive response (Choi et al., 2006; Chi et al., 2020; Poureini et al., 2025). Many researchers have utilized DO-stat feeding strategies to achieve high yields. For instance, a DO-stat fed-batch strategy was employed to produce *β*-carotene using an engineered *Yarrowia lipolytica* strain, resulting in a final yield of 2.01 g/L, which was 1.28 times that of standard fed-batch fermentation (Lv et al., 2020). Similarly, introducing a two-variable DO-stat strategy in the fermentation of *Pichia pastoris* for rGuamerin production resulted in expression levels 40% higher than manually fed batches (Lim et al., 2003). In the operation of this strategy, the initiation of substrate feeding depends on the control system’s accurate determination of a sharp rise in DO. However, in practice, the DO baseline under substrate-sufficient conditions changes dynamically due to factors such as the rheological properties of the broth, oxygen transfer efficiency, and the response characteristics of the O2 sensor. This necessitates that operators adjust the DO threshold for triggering feeding in real-time according to baseline variations. Consequently, the stable operation of the DO-stat strategy places high demands on the experience and knowledge of the personnel. Improper control parameter settings can easily lead to excessive or insufficient substrate feeding, thereby affecting process stability. Therefore, developing a fully automated control system capable of adaptively adjusting DO-stat control parameters based on cellular metabolic characteristics and real-time reactor DO changes is essential for ensuring the stability of the *E. coli* recombinant protein expression process.

To construct a fully automated system capable of adaptive parameter adjustment, it is first necessary to utilize computer algorithms to replace human operators in accurately identifying DO baseline values from complex DO fluctuation patterns. In recent years, pattern recognition technologies based on machine learning and artificial intelligence (AI) have provided strong technical support for this goal (Cheng et al., 2023; Huang et al., 2025). For example, the construction of soft sensors and predictors for glycerol and 1,3-propanediol based on artificial neural network models, combined with advanced feed controllers, increased the yield of 1,3-propanediol produced by *Clostridium butyricum* to 64.39 g/L (Huang et al., 2023). In this study, using AI algorithms to identify baseline changes in DO during *E. coli* cultivation represents a potentially effective approach.

This study employs a recombinant *E. coli* strain expressing Superfolder Green Fluorescent Protein (sfGFP) as a model system to systematically investigate the effects of induction time, inducer dosage, and different DO-stat control parameter combinations on cell growth and sfGFP synthesis. Subsequently, an Artificial Neural Network (ANN) model was constructed to identify dynamic changes in the DO baseline online, integrating the recognition results into the decision-making process of the DO-stat feeding strategy. Simultaneously, automatic control algorithms were developed for aeration, agitation speed, inducer addition, induction temperature regulation, and nitrogen source feeding. These were integrated with the glucose feeding control algorithm to ultimately achieve fully automated, unattended control of the sfGFP expression process in recombinant *E. coli*.

## 2 Materials and methods

### 2.1 Microorganism and cultivation process

#### 2.1.1 Strain

An *E. coli* BL21(DE3) strain expressing sfGFP was used. It was stored in 25% glycerol tubes at −70 °C.

#### 2.1.2 Media

Solid activation medium (g/L): Yeast extract (5), Tryptone (10), NaCl (5), Agar (20). Liquid seed medium (g/L): Yeast extract (5), Tryptone (10), NaCl (10). Fermentation medium (g/L): Glucose (10), Yeast extract (4), KH_2_PO_4_ (13.5), (NH_4_) _2_HPO_4_ (4), Citric acid monohydrate (2), MgSO_4_ (anhydrous) (0.97), Trace element solution (10 mL). Trace element solution (g/L): FeSO_4_·7H_2_O (10), CuSO_4_·5H_2_O (3), MnSO_4_·4H_2_O (0.5), ZnSO_4_·7H_2_O (5.25), (NH_4_)_6_Mo_7_O_24_ (0.1), Na_2_B_4_O_7_·10H_2_O (0.2), CaCl_2_ (2). (Dissolved in 83.3 mL of 36–38% HCl and diluted to 1 L). Feeding medium (g/L): Glucose (500), Yeast extract (100).

#### 2.1.3 High-density cultivation operation

*Escherichia coli* stored at −80 °C was inoculated onto LB solid medium for activation and cultured at 37 °C for 18 h. A ring of colonies was picked from the activated medium and inoculated into a 500 mL shake flask containing 100 mL of LB liquid medium. This seed culture was incubated at 37 °C and 200 rpm for 10 h. The seed culture was then inoculated at 3% (v/v) into a 1.5 L bioreactor (Cloud Ready™, TJX Bioengineering, China) containing 0.7 L of fermentation medium. Initial culture conditions were set as follows: agitation speed 400 rpm, aeration rate 0.5 L/min, and temperature 37 °C. Throughout the fermentation process, pH was maintained at 7.0 by adding ammonia water (28%). Depending on the specific experimental design, agitation and aeration were regulated via either manual or automatic control modes. In manual mode, when DO dropped below 20%, aeration was increased by 0.5 L/min and agitation by 200 rpm, up to process maximums of 800 rpm and 1.5 L/min, respectively. In automatic mode, these parameters were adjusted according to the control algorithm described in Section 2.4 (2). Induction was initiated by adding a specified amount of isopropyl-*β*-D-thiogalactopyranoside (IPTG) at a specific time point, after which the temperature was lowered to 28 °C. Induction operations were also performed in either manual or automatic modes (algorithm described in Section 2.4 (4)). During cultivation, when glucose depletion caused a sharp rise in DO, the DO-stat glucose feeding strategy was triggered. In manual mode, the DO thresholds for starting/stopping feeding and the feed rate were manually adjusted based on real-time culture characteristics. Once glucose feeding started, yeast extract was fed at a constant rate of 1.0 g/L/h to supplement nitrogen. In automatic mode, these feeding operations were executed by the computer via the algorithm in Section 2.4 (3).

#### 2.1.4 Optimization of induction conditions

An orthogonal experimental design was employed to optimize process parameters during the induction phase. An *L*_9_(3^4^) orthogonal array was selected to investigate the effects of induction start time (IT), IPTG addition amount (IA), and the feed start/stop threshold (SSH) in the DO-stat strategy on sfGFP expression. Range analysis was used to analyze the orthogonal experiment data to clarify the order of importance and the optimal level combination for each factor, as shown in Equations 1, 2. This method quantifies the contribution of each factor to the response variable by calculating the mean (*k*) and range (*R*) at different levels (Chai et al., 2021). By comparing *k_ij_* values within the same factor, the optimal level is determined. The magnitude of *R_j_* directly reflects the extent of the impact of level changes on the results: a larger *R_j_* indicates a more significant effect on sfGFP expression.


$$k_{\mathrm{ij}}=\frac{K_{\mathrm{ij}}}{s}=\frac{1}{s}\sum_{m=1}^{s}Y_{\mathrm{jm}}$$ (1)

$$R_{j}=\max(k_{1j},k_{2j},\cdots k_{\mathrm{ij}})-\min(k_{1j},k_{2j},\cdots k_{\mathrm{ij}})$$ (2)

Where *j* represents the factor, *i* represents the level, *s* is the number of repetitions for each level, *Y_jm_* is the experimental observation at that level, *k_ij_* represents the mean value of factor *j* at level *i*, and *R_j_* represents the difference between the maximum and minimum mean values for factor *j*.

### 2.2 Analytical methods

#### 2.2.1 Biomass (OD_600_) detection

The absorbance of the fermentation broth was measured at 600 nm using a spectrophotometer (V-1100D, Shanghai Meipuda Instrument Co., Ltd.) to obtain the OD_600_ value, which correlates with bacterial cell concentration.

#### 2.2.2 Glucose concentration detection

Glucose concentration (g/L) was detected using a biochemical analyzer (CG220 Bio-process Analyzer, Beijing CELL GENE Co., Ltd.).

#### 2.2.3 sfGFP fluorescence intensity detection

The fermentation broth was centrifuged to separate the cell pellet and supernatant, and the fluorescence intensity of both fractions was measured (Cellular Flu. Intensity and Supernatant Flu. Intensity). The cell pellet and supernatant were each diluted to appropriate gradients. Using black-walled, clear-bottom 96-well plates, the samples were placed in a microplate reader (SpectraMax Mini, Shanghai Molecular Devices Co., Ltd.) to measure the fluorescence intensity of the cells and supernatant at an emission wavelength of 485 nm and an excitation wavelength of 525 nm. The specific fluorescence intensity (Specific Flu. Intensity), representing the sfGFP expression capacity normalized to biomass, was calculated (Equation 3).


$$\text{Specific}\;\mathrm{Flu}.\text{Intensity}=\frac{\text{Cellular}\;\mathrm{Flu}.\text{Intensity}}{\text{biomass}(OD_{600})}$$ (3)

### 2.3 ANN recognition model construction

**Figure 1:**
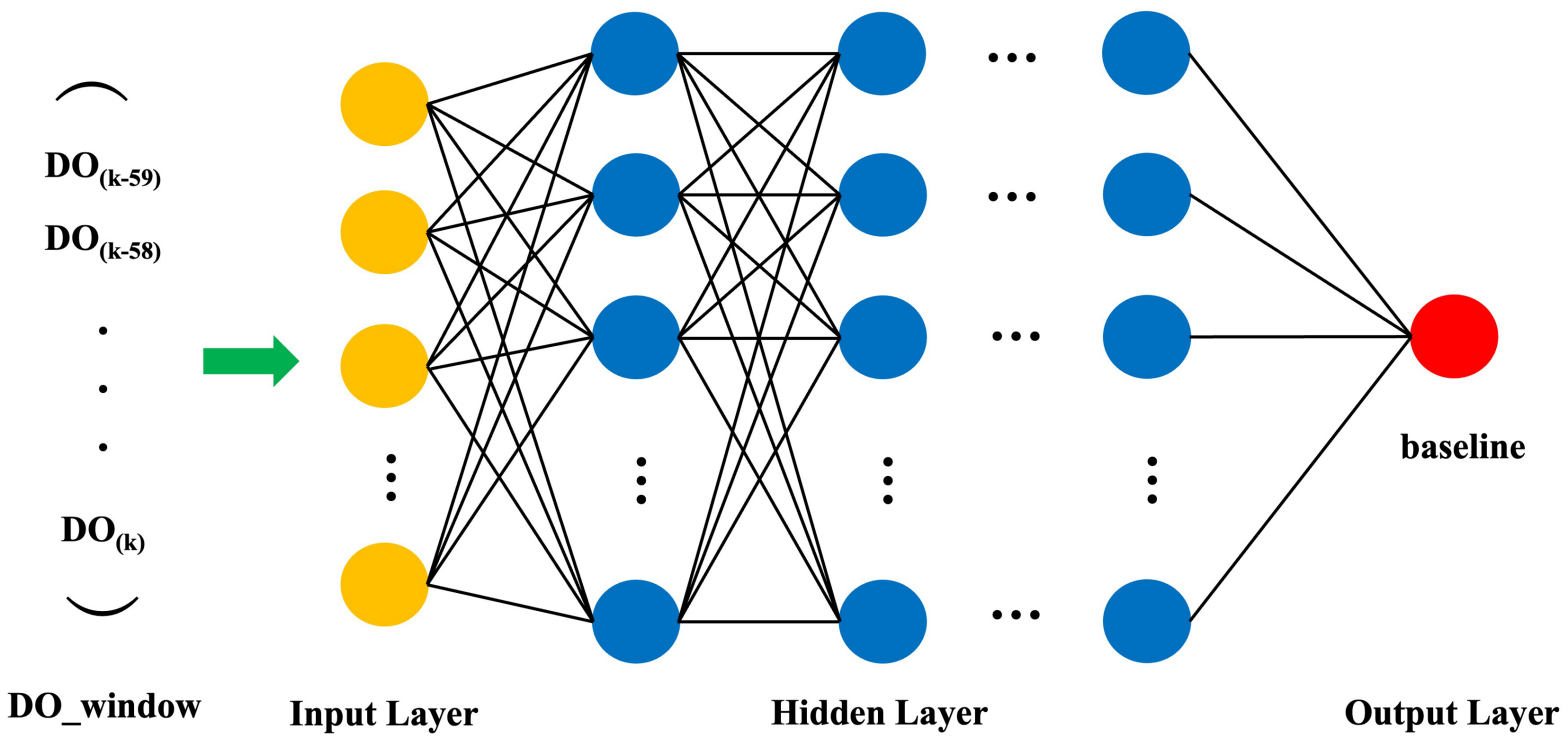
Architectural topology of the MLP neural network model for DO baseline recognition. The model comprises an input layer (60 neurons representing the DO_window), four fully connected hidden layers (1,024, 512, 256, and 128 neurons, respectively), and a single-neuron output layer delivering the predicted baseline value.

An ANN model was developed for DO baseline recognition using a Multi-Layer Perceptron (MLP) architecture (Rumelhart et al., 1986). The input layer comprises 60 neurons, processing a sliding window of the 60 most recent DO data points (DO_window). The architecture includes four fully connected hidden layers containing 1,024, 512, 256, and 128 neurons, respectively. The output layer contains one neuron representing the baseline value at the current time point (Figure 1). Neurons between layers are connected as shown in Equation 4, utilizing the ReLU function (Wang et al., 2023; Zhao et al., 2025) as the activation function to enhance the model’s non-linear fitting capability.


$$f=F(w_{i}x_{i}+b_{i})$$ (4)

Where *f* is the activation function, *w_i_* is the weight vector, *x_i_* is the input vector (output from the previous layer), and *b_i_* is the bias vector.

To train the final model, data were normalized and split into training and testing sets at a 1:1 ratio. Mean Squared Error (MSE) was used as the loss function (Equation 5).


$$L\mathrm{oss}=\frac{1}{n}\sum_{i=1}^{n}{\left(Y_{\text{experiment},i}-Y_{\text{prediction},i}\right)}^{2}\;\;\;$$ (5)

Where *Y_experiment,i_* is the measured value and *Y_prediction,i_* is the model-predicted value.

The gradient descent algorithm (Bottou, 2010) was employed for training, implemented using the PyTorch framework. Training was performed in a GPU-accelerated environment using the Adam optimizer for parameter iteration to minimize the Loss value.

### 2.4 Design of the unattended intelligent control system

**Figure 2:**
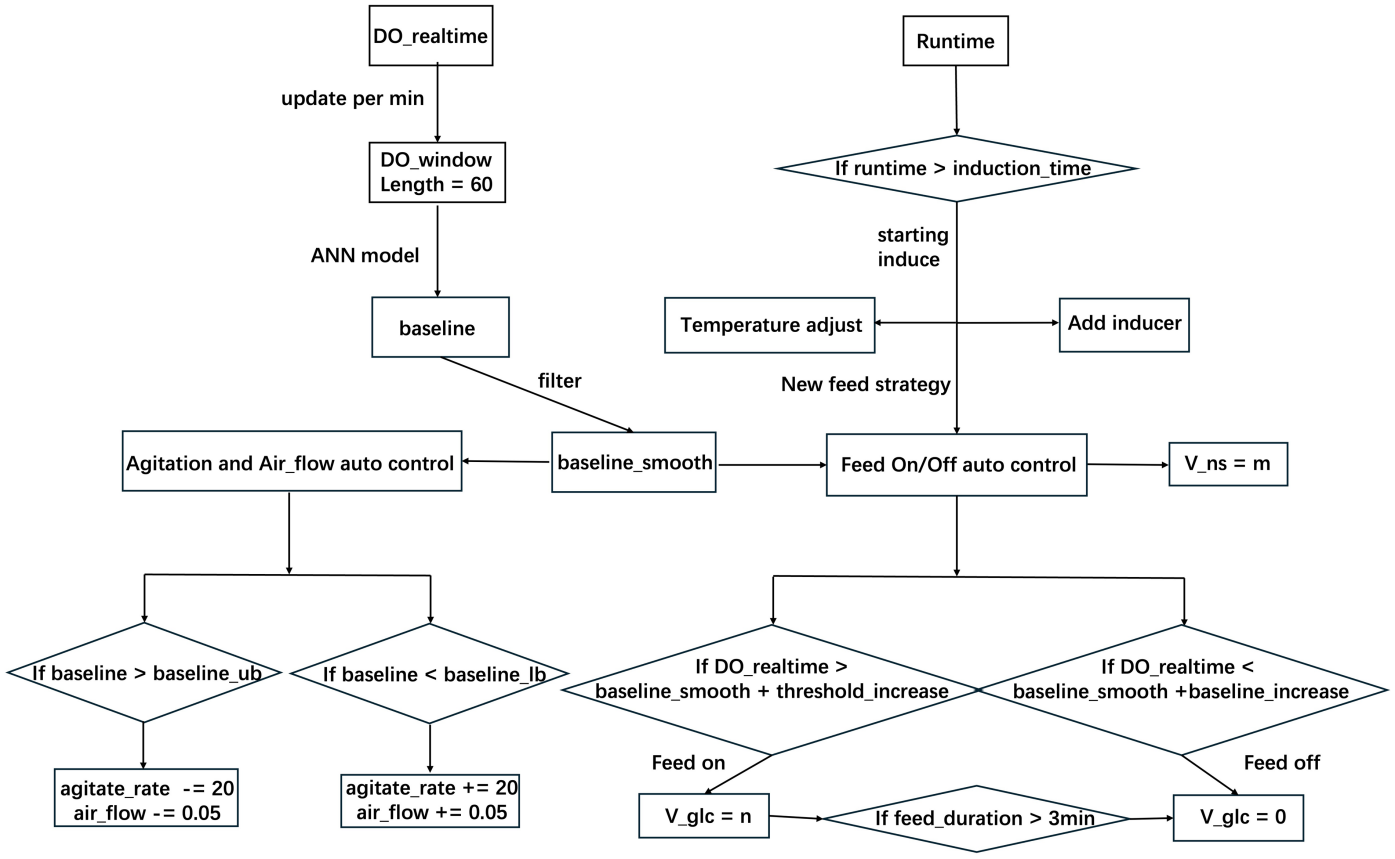
Schematic workflow and logic control of the NeuroStat-Ctrl intelligent system. The diagram illustrates the integration of real-time data acquisition, ANN-based baseline recognition (with Savitzky–Golay filtering), and automated control modules for aeration, agitation, carbon/nitrogen feeding, and time-triggered IPTG induction with concurrent temperature regulation.

Combining the baseline identified by the ANN model with the traditional DO-stat substrate feeding strategy, we constructed an adaptive control parameter adjustment strategy. This was integrated with automatic control mechanisms for agitation rate, aeration, inducer addition, and nitrogen source feeding to design the unattended intelligent control system shown in Figure 2, named NeuroStat-Ctrl. Developed in Python, the system runs on the host computer of the bioreactor and communicates with the reactor via the OPC-UA standard interface for real-time data read/write and process parameter control. The main functional modules include:

(1) Real-time Data Acquisition and DO Baseline Recognition: The DO_realtime value was acquired every second and stored in a sliding time window at 1 min intervals. The time window (DO_window) length was set to 60, meaning it always contained the most recent 1 h of DO data, updated every minute. After each update, the current time window was input into the trained ANN model for baseline recognition. A Savitzky–Golay filter (Jiang et al., 2019) was applied online to the raw baseline data (baseline) to generate a smoothed baseline (baseline_smooth), serving as the core reference for subsequent intelligent control.
(2) Automatic Control of Oxygen Supply Conditions: After setting initial agitation rate (agitate_rate) and aeration rate (air_flow) and completing inoculation, the system automatically adjusted agitate_rate and air_flow based on the baseline_smooth value updated every minute. When baseline_smooth fell below the lower limit (baseline_lb), the system judged the fermentation broth to be oxygen-deficient and increased agitate_rate by 20 rpm and air_flow by 0.05 L/min. In the mid-to-late fermentation phase, with constant oxygen supply conditions, DO would continuously rise as cellular metabolic activity declined. When baseline_smooth exceeded the upper limit (baseline_ub), the system judged the fermentation broth to be oxygen-excessive and decreased agitate_rate by 20 rpm and air_flow by 0.05 L/min.
(3) Automatic Feeding of Carbon and Nitrogen Sources: The system updated baseline_smooth every minute. When DO_realtime rose above baseline_smooth + threshold_increase, the system judged that glucose in the broth was depleted and triggered glucose feeding, adding glucose at a preset rate (V_glc). As glucose was added, DO_realtime continuously decreased. When DO_realtime fell below baseline_smooth + baseline_increase, glucose feeding stopped. To prevent overfeeding due to DO response delay, NeuroStat-Ctrl implemented a safety rule: if glucose feeding continued for a full 3 min, it was forcibly stopped regardless of whether DO_realtime had dropped below baseline_smooth + baseline_increase. Once glucose feeding was triggered for the first time, constant-rate nitrogen source feeding was automatically initiated simultaneously at a fixed rate (V_ns) of 1.0 g/L/h.
(4) Automatic Induction Operation: The NeuroStat-Ctrl system automatically executed the induction operation based on the actual fermentation time (Runtime) recorded by the fermenter. When Runtime exceeded the set induction_time, the system automatically activated a peristaltic pump to add the inducer IPTG and simultaneously adjusted the cultivation temperature to 28 °C. For the cell growth phase and induction phase of *E. coli* high-density cultivation, NeuroStat-Ctrl automatically loaded different control parameter sets: baseline_increase, threshold_increase, and V_glc.

## 3 Results and discussion

### 3.1 sfGFP expression performance under traditional DO-stat glucose feeding strategies

**Figure 3:**
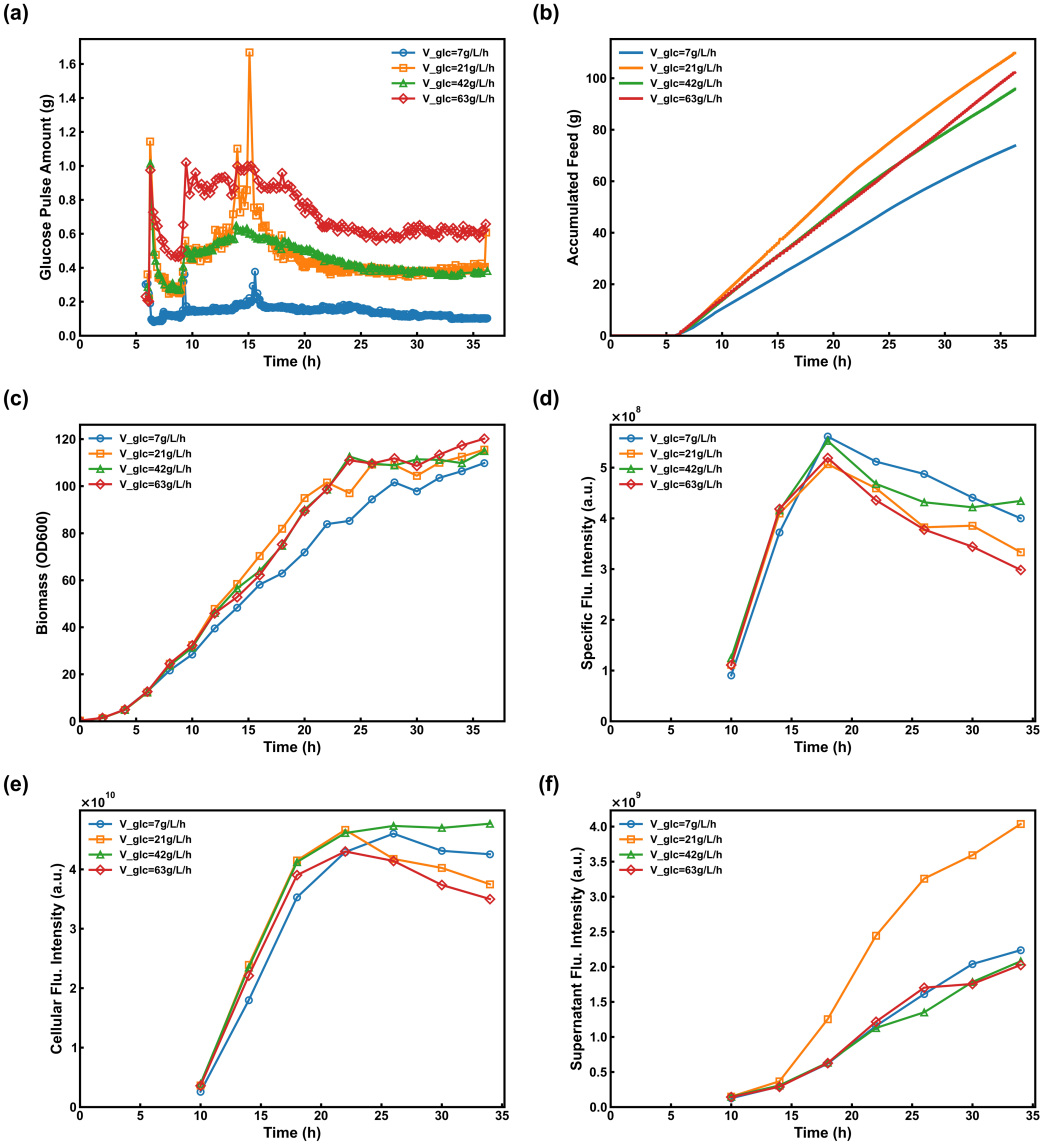
Fermentation performance under traditional DO-stat strategies with varying V_glc_ (Batches #1–#4). **(a)** Individual glucose pulse volumes over time; **(b)** Cumulative glucose consumption; **(c)** Biomass accumulation (OD_600_); **(d)** Specific fluorescence intensity; **(e)** Cellular fluorescence intensity; and **(f)** Supernatant fluorescence intensity.

DO-stat is a widely employed glucose feeding strategy designed to maintain residual glucose at a limiting concentration. This study initially evaluated the control performance of this strategy during high-density cultivation of the recombinant strain and its subsequent effects on sfGFP expression. Four fermentation batches were executed using the DO-stat protocol, with the DO thresholds for starting and stopping feeding set at 65 and 25%, respectively. Substrate was supplied at a fixed feed rate (V_glc) upon each trigger. For Batches #1—#4, V_glc was established at 7.0, 21.0, 42.0, and 63.0 g/L/h, respectively. In all instances, glucose was administered in discrete pulses, with individual pulse volumes depicted in Figure 3a. Batch #1 exhibited the minimum pulse volume alongside the maximum triggering frequency. Conversely, Batch #4 displayed the largest pulse volumes—peaking at approximately 0.9 g during the mid-phase before declining to 0.6 g in the later stages—thereby resulting in the lowest triggering frequency. Batches #2 and #3 maintained relatively consistent pulse volumes and triggering periodicities. Analytical measurements confirmed the absence of glucose accumulation throughout the feeding phase across all batches, with residual concentrations remaining near 0.0 g/L. The profiles for cumulative glucose consumption, biomass accumulation, and specific fluorescence intensity are illustrated in Figures 3b–d, respectively. A clear correlation between glucose feeding dynamics and sfGFP expression levels was observed:

(1) Batch #1 featured the minimum V_glc setpoint, characterized by the smallest individual pulse volumes and the highest triggering frequency. However, the total cumulative glucose consumption was the lowest among all cohorts, which proved insufficient to satisfy the metabolic demand for rapid proliferation, leading to a diminished biomass concentration. Interestingly, during the induction phase, this restricted glucose supply effectively circumvented the repressing effects of substrate accumulation on sfGFP expression. Consequently, Batch #1 maintained a superior specific fluorescence intensity throughout most of the process. Nonetheless, due to the biomass limitation, the final cellular fluorescence intensity (4.48 × 10^10^) remained slightly below that of Batch #3 (Figure 3e).
(2) Batch #2 utilized a higher V_glc setpoint and consumed more total glucose than Batch #1. Notably, a control malfunction occurred between 14 and 16 h of cultivation. Although feeding initiated at the 65% DO threshold, an abnormally delayed DO response prevented the system from reaching the 25% termination threshold promptly, resulting in an excessive single pulse of 1.6 g. This transient overfeeding significantly inhibited sfGFP synthesis. As illustrated in Figure 3f, the supernatant fluorescence intensity spiked after 15 h, eventually surpassing Batches #1, #3, and #4 by 80.5%, 94.2%, and 99.2%, respectively. This phenomenon suggests that excessive glucose prompted metabolic overflow and potential cell lysis, leading to the substantial leakage of intracellular sfGFP into the extracellular medium.
(3) In Batch #3, despite a higher V_glc setpoint compared to Batch #2, the DO-stat strategy remained robust, with no operational failures observed. This resulted in a slightly lower cumulative glucose consumption than Batch #2, yet the balance between pulse volume and frequency facilitated optimal sfGFP biosynthesis. The specific fluorescence intensity remained stable without significant degradation toward the end of the process, ultimately exceeding that of Batch #1 (Figure 3d). Combined with its high biomass accumulation, this batch achieved the highest overall sfGFP expression level among all experimental groups, reaching 4.97 × 10^10^ (Figure 3e).
(4) Batch #4, governed by the highest V_glc setpoint, exhibited the largest individual pulse volumes coupled with the lowest triggering frequency. Although its total glucose consumption mirrored that of Batch #3, the high-amplitude pulses induced localized glucose toxicity, which hindered sfGFP synthesis and protein folding. As a result, this batch yielded the lowest final specific fluorescence (3.08 × 10^8^) and cellular fluorescence intensity (3.71 × 10^10^) across the study (Figures 3d,e).

Based on the outcomes of these four batches, several key conclusions can be synthesized:

(1) Impact of Feeding Dynamics on Expression: Under the DO-stat regime, recombinant protein expression levels are determined not only by the total cumulative glucose consumption but also by the specific dynamics of the feeding pulses. Specifically, the synergy between individual pulse volumes and their triggering frequencies is a critical determinant of expression efficiency, where moderate values in both parameters facilitate optimal protein synthesis.
(2) Limitations of Fixed-Threshold Strategies: During high-density *E. coli* cultivation, the DO baseline undergoes continuous and complex fluctuations driven by the interplay between oxygen supply (aeration and agitation) and the cellular oxygen uptake rate. Consequently, traditional DO-stat strategies—characterized by fixed start/stop thresholds and a static V_glc—lack the flexibility to adapt to these variations, making it difficult to maintain feeding parameters within an ideal operational range throughout the process.
(3) Phase-Specific Metabolic Requirements: The two distinct phases of cultivation demand contrasting feeding intensities: the growth phase requires a robust glucose supply to support rapid biomass accumulation, whereas the induction phase necessitates a strictly regulated and reduced feeding rate. This reduction is essential to mitigate the detrimental effects of metabolic overflow caused by excess substrate, thereby ensuring an environment conducive to high-efficiency recombinant protein expression.

### 3.2 Optimization of induction conditions

**Figure 4:**
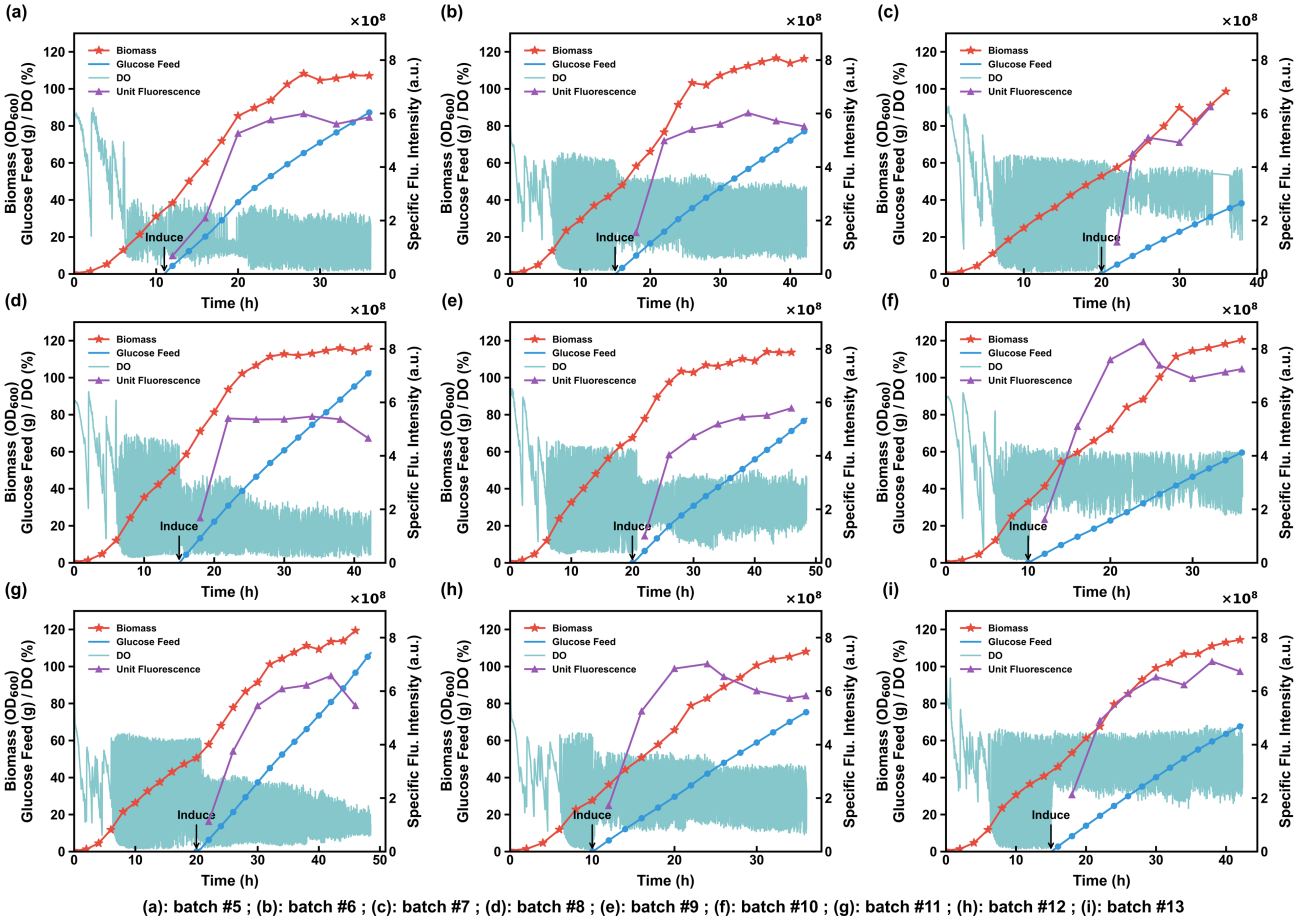
Dynamic fermentation profiles of the nine orthogonal experimental batches (#5–#13). Each panel displays the time-course correlations between biomass, glucose feed rate, DO fluctuations, and specific fluorescence intensity under different induction and feeding regimes.

Based on preliminary findings, the optimal conditions required for the cell growth phase and the induction phase differ significantly. Building on Section 3.1, the induction conditions for sfGFP expression in the model strain were further systematically optimized. Induction start time (IT), inducer addition amount (IA), and start/stop threshold (SSH) were selected as key factors. A three-factor, three-level orthogonal experiment was designed, with specific results shown in Table 1. To ensure consistent and moderate glucose supply and cultivation state during the growth phase, all experimental batches uniformly used a glucose feeding start threshold of 60%, stop threshold of 25%, and a glucose feeding rate (V_glc) of 21.0 g/L/h during growth. According to the orthogonal design, nine fermentation batches were performed, numbered #5 to #13, with corresponding results shown in Figure 4.

**Table 1:** Design and results of the orthogonal experiment (*L*_9_(3^4^)) with three factors and three levels for the optimization of sfGFP induction conditions.

Batch	IT (h)	IA (mmol/L)	SSH (%/%)	Specific Flu. Intensity (au)
#5	10	0.1	15/10	6.08 × 10^8^
#6	15	0.1	30/25	5.71 × 10^8^
#7	20	0.1	50/45	6.36 × 10^8^
#8	15	0.3	15/10	4.92 × 10^8^
#9	20	0.3	30/25	5.89 × 10^8^
#10	10	0.3	50/45	7.52 × 10^8^
#11	20	0.5	15/10	5.60 × 10^8^
#12	10	0.5	30/25	6.30 × 10^8^
#13	15	0.5	50/45	7.04 × 10^8^
*k*_1_	6.63 × 10^8^	6.05 × 10^8^	5.53 × 10^8^	
*k*_2_	5.89 × 10^8^	6.11 × 10^8^	5.97 × 10^8^	
*k*_3_	5.95 × 10^8^	6.31 × 10^8^	6.98 × 10^8^	
*R*	7.47 × 10^7^	2.66 × 10^7^	1.44 × 10^8^	

Range analysis was performed on the specific fluorescence intensity data from the nine batches to clarify the degree of influence of each process parameter on the fermentation outcome. Analysis of the range (*R*) values for each factor (Table 1) shows: *R*(SSH) > *R*(IT) > *R*(IA). This result indicates that during the sfGFP induction phase, SSH is the most critical factor affecting expression level, followed by IT, while IA has a relatively weaker influence, showing a wider operational tolerance. From the trend of mean values (*k*) at different levels, the means for SSH show a significant increasing trend: *k*₁ < *k*₂ < *k*₃, suggesting that setting SSH to the higher value of 50%/45% is most favorable for sfGFP expression. The means for IT show the trend *k*₁ > *k*₃ > *k*₂, indicating that an earlier induction start time is more beneficial. For IA, the means show only a slight increasing trend *k*₁ < *k*₂ < *k*₃, reflecting the smallest impact of this parameter on sfGFP expression. Based on mean analysis, the theoretically optimal induction conditions were determined as: IT = 10.0 h, IA = 0.5 mmol/L, SSH = 50%/45%.

**Figure 5:**
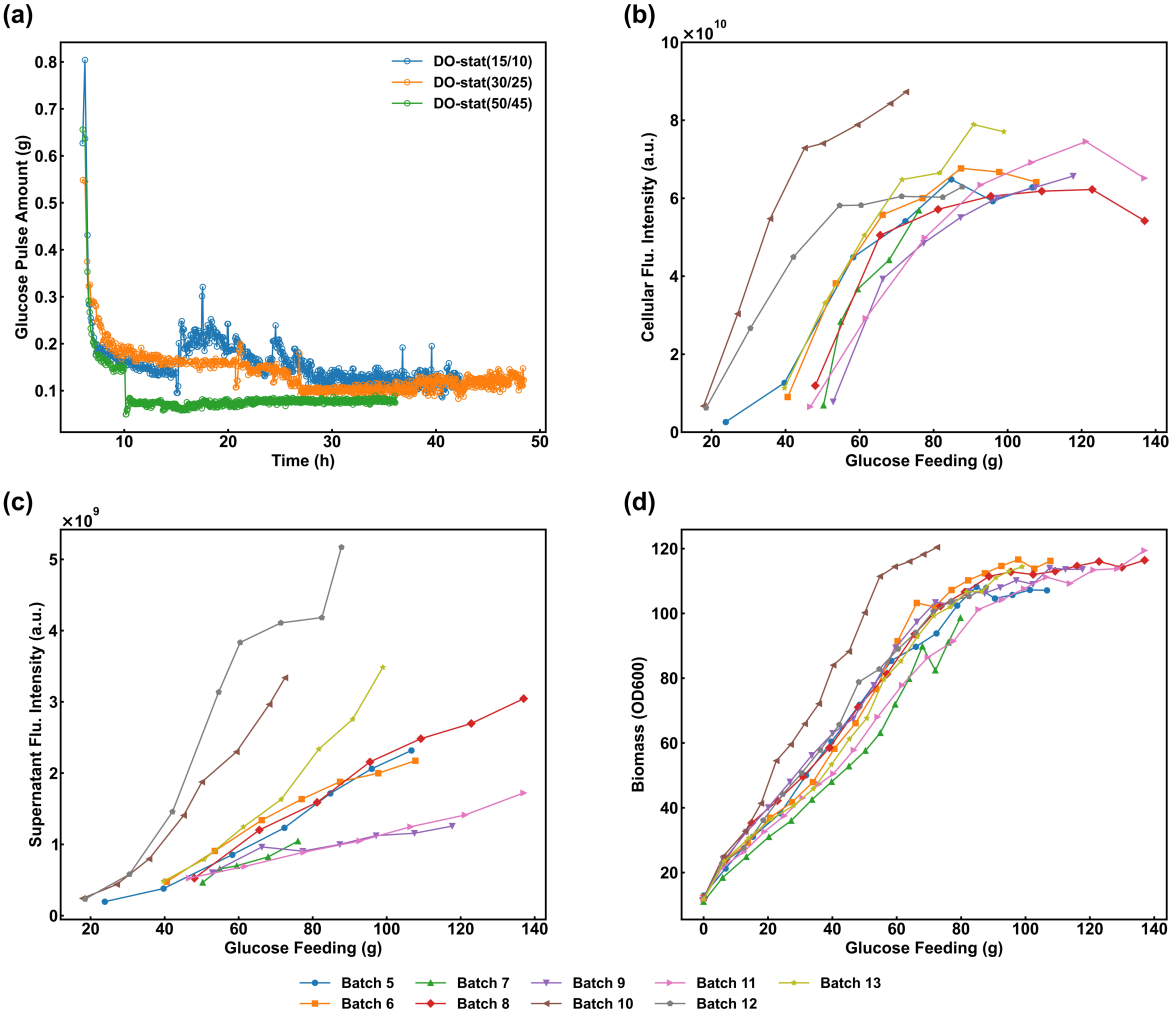
Influence of start/stop thresholds (SSH) on metabolic and expression parameters. **(a)** Correlation between SSH and glucose pulse volumes; **(b,c)** Relationship between cumulative glucose feeding and cellular/supernatant fluorescence; **(d)** Impact of glucose supply intensity on biomass accumulation.

Using batches #8 (SSH = 15%/10%), #9 (SSH = 30%/25%), and #10 (SSH = 50%/45%) as case studies, the effect of SSH on cell growth and sfGFP expression is discussed. During DO-stat control in the induction phase, the SSH setting directly determines the glucose supply intensity. As shown in Figure 5a, as the SSH setting increased, the single glucose pulse amount decreased correspondingly, and the feeding interval shortened. Comparing the cumulative glucose added during the entire induction phase for the three batches (Figures 4d–f) showed that batch #8 had the highest cumulative addition, batch #9 was intermediate, and batch #10 the lowest. Batch #10 achieved the highest biomass (OD₆₀₀ of 120), indicating that a lower glucose feeding rate during induction is more favorable for cell growth (Figure 5d). Further analysis of sfGFP expression performance showed: in batch #8, the lower SSH setting led to excessive glucose addition and persistently low DO levels (Figure 4d), inhibiting sfGFP synthesis, with specific and cellular fluorescence intensities of only 4.92 × 10^8^ and 5.72 × 10^10^, respectively (Figure 4d, Figure 5b). In batch #10, the higher SSH setting effectively limited glucose feeding during induction, mitigating metabolic overflow while maintaining DO at a more ideal level, promoting efficient sfGFP expression, achieving higher specific and cellular fluorescence intensities of 7.52 × 10^8^ and 9.06 × 10^10^, respectively (Figure 4f, Figure 5b), its specific fluorescence intensity increased by 52.85% compared to Batch #8.

To examine the influence of induction timing, Batch #10 (IT = 10 h) was compared with Batch #13 (IT = 15 h), both utilizing high SSH thresholds. The earlier initiation of induction in Batch #10 resulted in specific and total fluorescence intensities that were 6.98% and 12.5% higher, respectively, identifying 10 h as the optimal induction start time for maximized expression performance.

Range analysis suggested that increasing IA level can promote sfGFP expression. However, at higher IA levels, a greater metabolic burden on cells is often observed (Dvorak et al., 2015). Comparing batch #9 (IA = 0.3 mmol/L) and batch #12 (IA = 0.5 mmol/L), batch #12 had higher IA and a more optimal induction time, yet its specific and total fluorescence intensities were only 7.0% and 1.73% higher than batch #9, respectively (Figures 4e,h, Figure 5b). However, the supernatant fluorescence intensity of batch #12 increased significantly, reaching the highest value among all batches (Figure 5c). This is speculated to be possibly due to excessive IA addition causing cell death, ultimately leading to significantly increased sfGFP content in the supernatant. Furthermore, given the high cost of IPTG, a lower dosage is economically preferable. Therefore, balancing total yield, process stability, and economic feasibility, we determined IA = 0.3 mmol/L to be the practical optimal concentration for the subsequent automated fermentation experiments.

### 3.3 ANN recognition model construction

As previously established, the reliance on fixed start/stop thresholds within the DO-stat strategy often fails to maintain glucose concentrations at the optimal physiological level for sfGFP expression. A promising solution involves the dynamic adjustment of these thresholds in response to real-time fluctuations in fermentation parameters. In manual operation, while experienced personnel can calibrate thresholds by observing shift in the DO baseline, this approach is labor-intensive and highly susceptible to human error. To transition from manual intervention to an autonomous, algorithm-driven system for adaptive threshold regulation, the primary prerequisite is the capacity to accurately extract the DO baseline using robust data analysis. Throughout the cultivation lifecycle, the DO profile exhibits intricate dynamic behaviors driven by the interplay between the system’s oxygen transfer capacity and the cellular oxygen uptake rate. Consequently, the precise identification of the baseline from these complex signals necessitates the development of a dedicated baseline recognition model.

**Figure 6:**
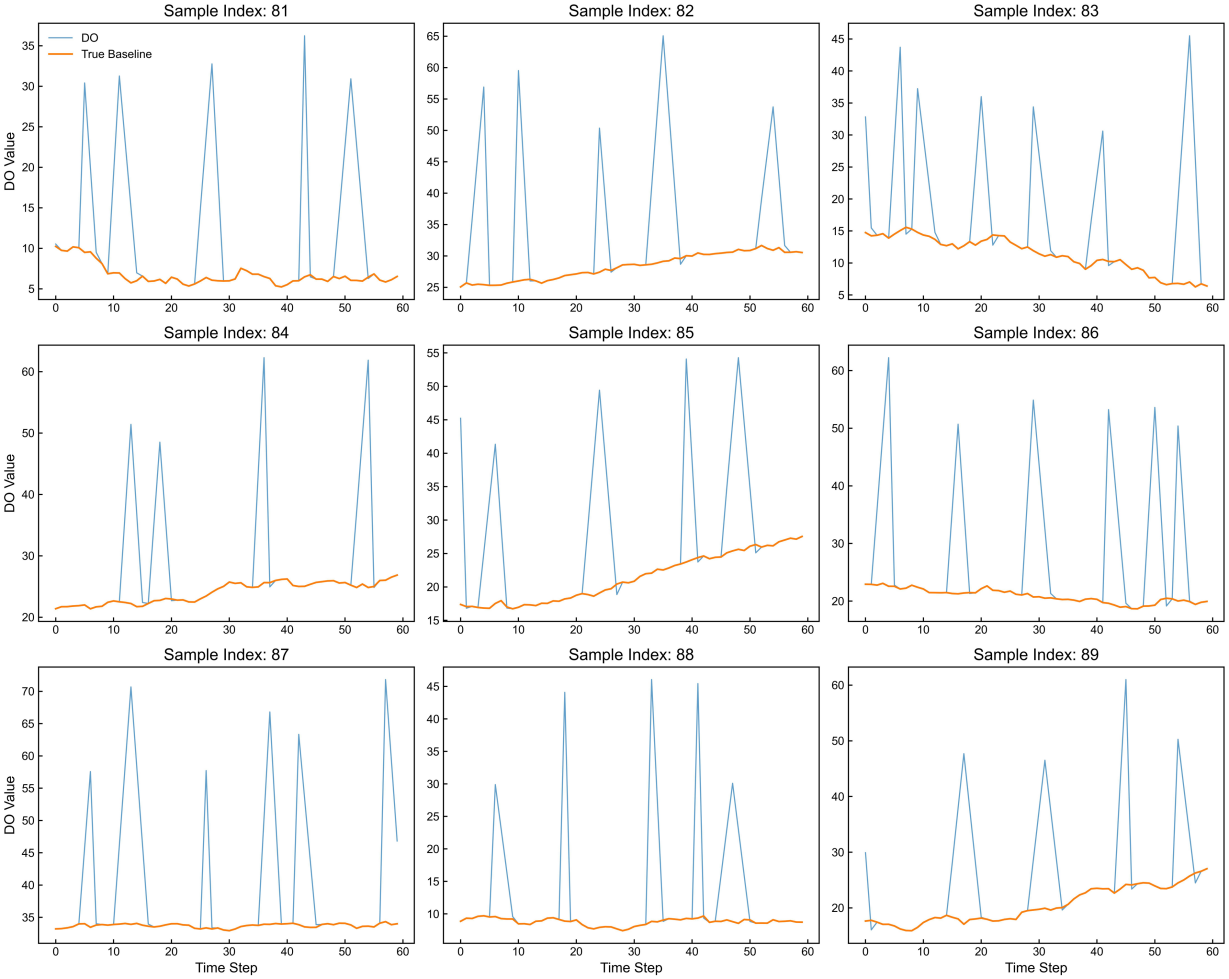
Representative examples of synthetic training samples generated by the data augmentation algorithm. Each sample includes a sequence of 60 DO observations (blue) and its corresponding ground-truth baseline value (orange) used to train the MLP model.

In recent years, machine learning has demonstrated substantial potential in time-series signal processing and complex nonlinear modeling (Zhang et al., 2020). Previous studies have successfully leveraged artificial neural network (ANN) models to tackle challenges in parameter soft-sensing and state recognition within various bioprocesses. In this study, a Multi-Layer Perceptron (MLP) model was developed, with its architectural topology illustrated in Figure 1. The effective training of such models necessitates an extensive dataset of labeled samples with predefined DO baseline values. However, because DO baselines cannot be directly recorded by physical sensors and require intensive manual annotation, acquiring a sufficient volume of high-quality training data from actual fermentation runs is inherently challenging. To circumvent this limitation, we developed a synthetic data generation algorithm modeled after the dynamic patterns observed during DO-stat operations. This algorithm produced a comprehensive dataset of 15,000 diverse training samples, capturing a wide array of fluctuation trends alongside their corresponding ground-truth baseline values. Each training instance comprised a sequence of 60 consecutive DO observations—serving as the feature input—and a single baseline value acting as the target label. Representative examples from this synthetic dataset are displayed in Figure 6.

**Figure 7:**
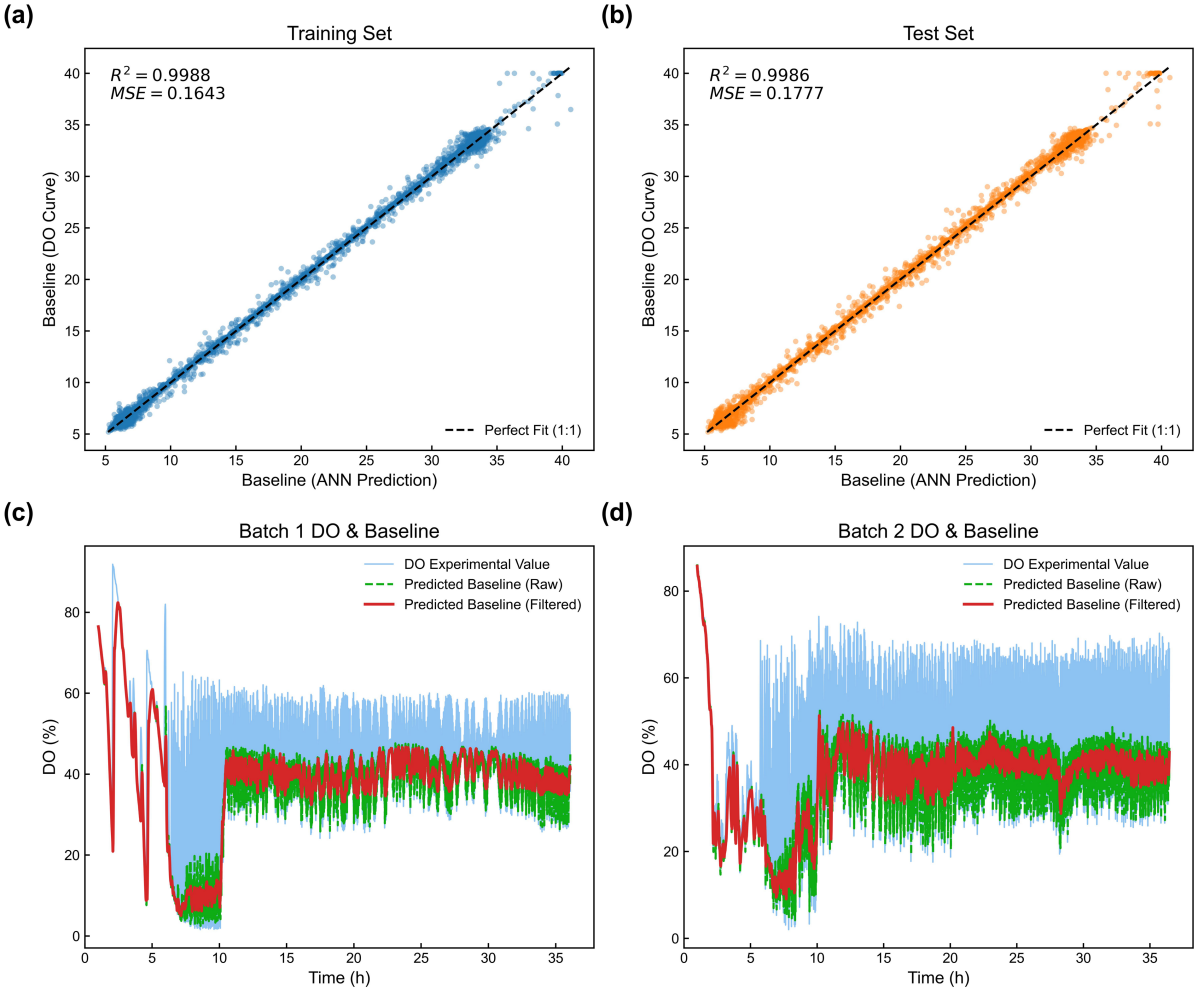
Predictive performance and experimental validation of the ANN model. **(a,b)** Regression analysis of predicted vs. target baseline values for training and test sets, including *R*^2^ and MSE metrics; **(c,d)** real-time baseline recognition (green) and Savitzky–Golay smoothing (red) applied to raw DO data (blue) from independent fermentation batches.

The synthetic dataset was partitioned into training and test sets using a 1:1 ratio. A training algorithm centered on gradient descent was implemented within the PyTorch framework, with model training conducted on a system equipped with dual NVIDIA GeForce RTX 4090 GPUs. Although the training process was accelerated using these high-performance GPUs, the computational cost for real-time inference remains minimal. During deployment, the baseline recognition step (executed once per minute) runs efficiently on an Intel Core i5-13500H CPU without requiring specialized hardware, ensuring the system’s compatibility with conventional industrial control computers. Both datasets were subsequently processed by the trained model to predict baseline values for each instance4. The correlation between predicted and ground-truth values is illustrated in Figures 7a,b. Specifically, the training set achieved an *R*^2^ of 0.9988 and a Mean Squared Error (MSE) of 0.1643, while the test set yielded an *R*^2^ of 0.9986 and an MSE of 0.1777. These metrics demonstrate the robust predictive accuracy of the MLP model when applied to algorithmically generated samples.

While the initial validation of the MLP model relied exclusively on synthetic data, its efficacy on real-world fermentation profiles required further substantiation. To ensure practical applicability, the model’s generalization capability was evaluated using experimental data. Raw DO data from two independent fermentation batches were organized into sliding windows (length = 60) and sequentially fed into the trained MLP model to predict baseline trajectories. The results are illustrated in Figures 7c,d, where the blue curve represents the raw DO data acquired by the sensor, and the green curve depicts the model-predicted baseline. Although the synthetic dataset is idealized, it comprehensively simulated actual DO-stat patterns, including sharp dissolved oxygen (DO) spikes following glucose depletion and subsequent declines or maintenance phases post-feeding. Additionally, the dataset encompassed diverse peak heights, widths, and frequencies, as well as various baseline trends (stable, decreasing, and increasing), which allowed the MLP model to capture the full spectrum of DO dynamics for accurate baseline recognition. Moreover, to bridge the “sim-to-real” gap—specifically to address the inevitable high-frequency sensor noise, signal delays, and electrode drift encountered in long-term fermentation—we integrated a Savitzky–Golay filter at the model’s output. The resulting filtered baseline (red curve) effectively eliminates high-frequency disturbances while retaining sensitivity to metabolic shifts. This demonstrates superior smoothness and robustness, fully meeting the stringent stability requirements for unattended intelligent control.

### 3.4 Construction and application of the NeuroStat-Ctrl system

The autonomous intelligent control system, NeuroStat-Ctrl (schematically illustrated in Figure 2), was developed and implemented for the automated regulation of aeration, agitation, glucose and nitrogen feeding rates, and IPTG induction during *E. coli* cultivation. Batch #14 served as the control, employing a manually adjusted DO-stat strategy with fixed start/stop thresholds of 60%/25% and a constant V_glc of 21.0 g/L/h throughout the process. In contrast, Batch #15 utilized NeuroStat-Ctrl for fully autonomous process control, with system parameters baseline_lb, baseline_ub, baseline_increase, threshold_increase, and V_glc configured to 25, 50, 15, 40, and 21 g/L/h, respectively. The feeding strategy during the induction phase remained identical to that of the control batch to ensure comparability.

**Figure 8:**
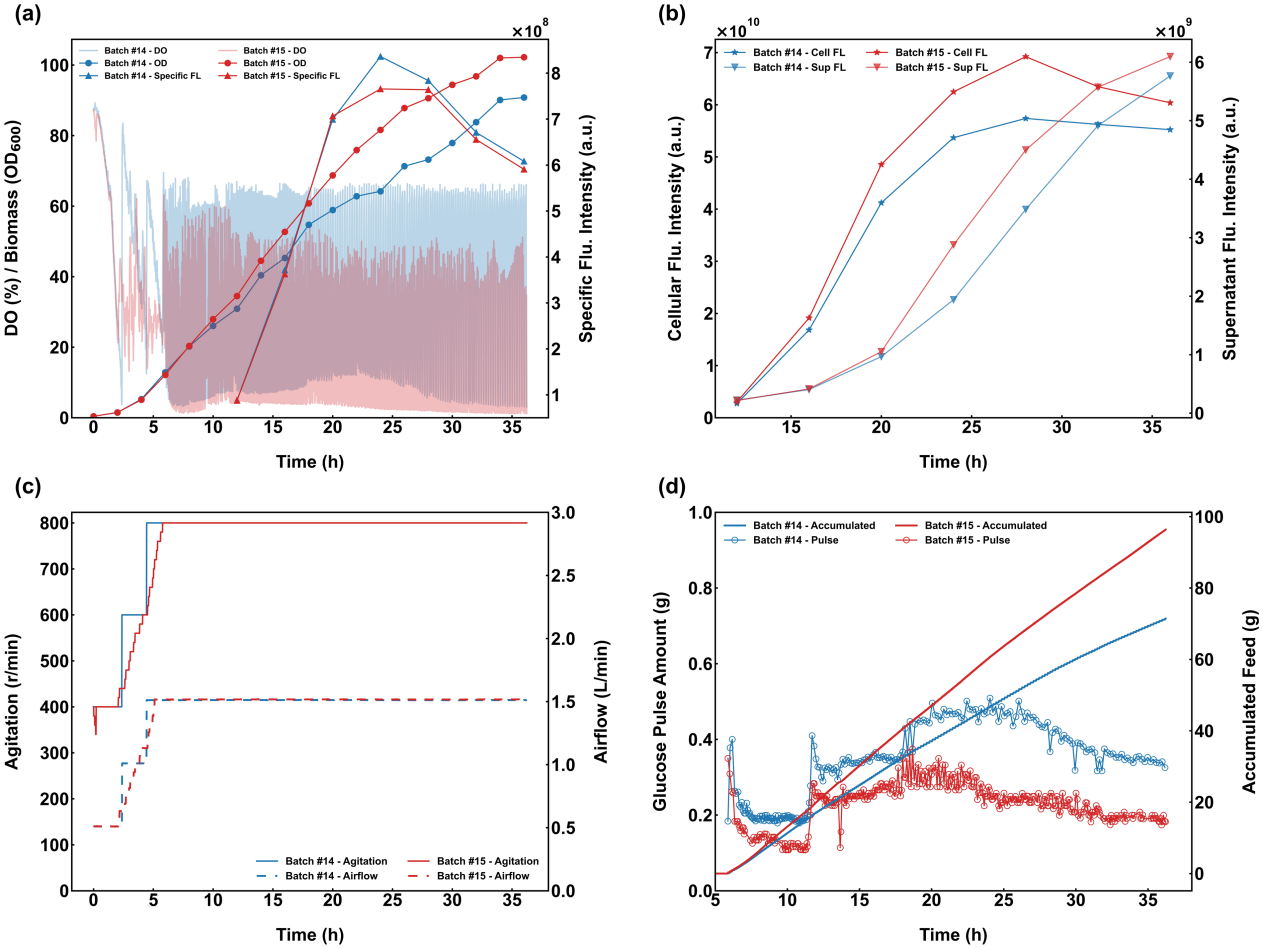
Comparative performance of the standard DO-stat (Batch #14) and the NeuroStat-Ctrl system (Batch #15). **(a,b)** Profiles of biomass, DO, and protein expression dynamics; **(c)** Comparison of manual vs. automated regulation of agitation and aeration; **(d)** Glucose pulse volumes and cumulative consumption.

The comparative fermentation performance of Batches #14 and #15 is presented in Figure 8. Beyond achieving unattended operation, the NeuroStat-Ctrl system demonstrated distinct process advantages: specifically, the adjustments to aeration and agitation in Batch #15 were significantly more gradual and refined during the early cultivation phase compared to Batch #14 (Figure 8c). Batch #15 was characterized by smaller individual pulse volumes but a higher triggering frequency, ultimately resulting in a higher cumulative glucose consumption than the manual control (Figure 8d). Despite the increased total glucose uptake, the granularity of the smaller pulse additions facilitated superior control stability. While specific fluorescence intensities were comparable between the two batches, the enhanced biomass concentration in Batch #15 resulted in a 9.32% increase in cellular fluorescence intensity relative to Batch #14 (Figure 8b).

**Figure 9:**
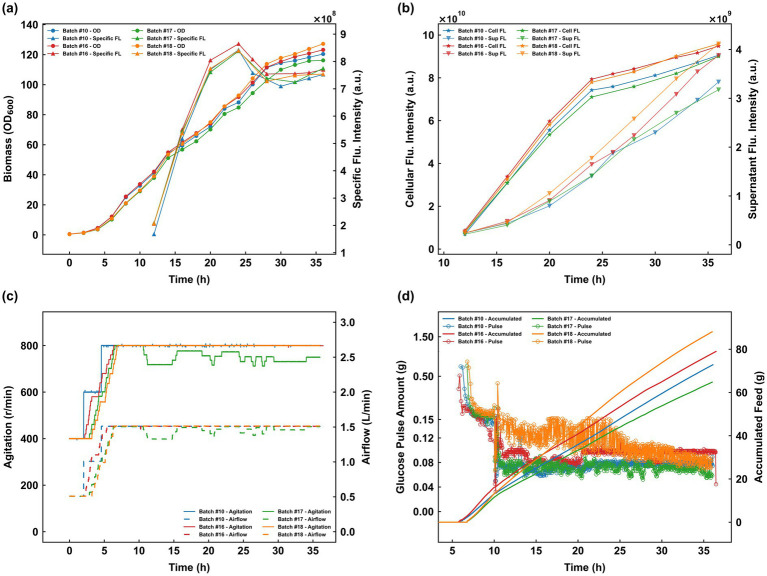
Evaluation of NeuroStat-Ctrl system robustness and adaptability under varied parameter configurations (Batches #16–#18) compared to the benchmark (Batch #10). **(a,b)** Profiles of biomass accumulation, specific fluorescence, and cellular/supernatant fluorescence dynamics; **(c)** Automated regulation of agitation and airflow according to cellular oxygen demand; **(d)** Comparison of individual glucose pulse volumes and cumulative glucose consumption.

To further evaluate system robustness, three additional experiments (Batches #16–#18) were conducted against Batch #10 as a benchmark (Figure 9). In Batch #16, the control parameters baseline_lb, baseline_ub, baseline_increase, threshold_increase, and V_glc were set to 25, 50, 15, 40, and 21 g/L/h, respectively; during the induction phase, baseline_increase, threshold_increase, and V_glc were adjusted to 5, 10, and 7 g/L/h, respectively. For Batch #17, the growth phase parameters were set to 25, 40, 15, 40, and 21 g/L/h, while the induction phase values for baseline_increase, threshold_increase, and V_glc were 0, 10, and 7 g/L/h, respectively. Batch #18 utilized growth phase parameters of 25, 50, 15, 40, and 21 g/L/h, with induction phase parameters of 0, 10, and 7 g/L/h. These diverse configurations resulted in distinct feeding profiles: Batches #16 and #18 exhibited higher cumulative glucose consumption and larger individual pulse volumes than Batch #10, whereas Batch #17 recorded the lowest cumulative feed with pulse amounts comparable to the benchmark (Figure 9d). Crucially, the specific fluorescence intensities of four batches were comparable, indicating that the refined feeding intensity provided by NeuroStat-Ctrl successfully averted the metabolic overflow observed in earlier batches (#14 and #15). Enhanced biomass levels in Batches #16 and #18 led to 4.71% and 5.87% increases in cellular fluorescence intensity, respectively, over Batch #10 (Figures 9a,b). Most notably, by fine-tuning the induction-phase strategy, Batch #18 achieved remarkable gains, with specific and cellular fluorescence intensities increasing by 27.62% and 58.84%, respectively, relative to Batch #15. Furthermore, by setting the baseline_ub to 40 in Batch #17, the system automatically modulated agitation and aeration in response to cellular oxygen demand, maintaining the DO baseline precisely within the target range (Figure 9c). These consistent outcomes across varied configurations underscore the exceptional adaptability and precision of the NeuroStat-Ctrl system.

## 4 Conclusion

This study analyzed the impact of different DO-stat strategies on the key issue of overflow metabolism during *E. coli* fermentation for production, developed an optimized *E. coli* DO-stat fermentation process, and achieved significant improvements in cell density and protein yield. Simultaneously, an artificial intelligence-based fully automated DO-stat control system for *E. coli* was proposed, effectively addressing some shortcomings of traditional fixed-threshold DO-stat control. With the support of the fully automated control system, the entire *E. coli* fermentation production successfully achieved high-density cultivation and high-efficiency product expression in a fully unattended manner. These results confirm that combining data-driven neural network models with bioprocess engineering optimization can effectively solve stability challenges in high-throughput process development within parallel bioreactors, providing a universal and efficient solution for the intelligent manufacturing of recombinant proteins.

## Data Availability

The raw data supporting the conclusions of this article will be made available by the authors, without undue reservation.
